# C-Reactive Protein-to-Lymphocyte Ratio as a Prognostic Biomarker in Acute Ischemic Stroke Patients Undergoing Mechanical Thrombectomy: A Multicenter Study

**DOI:** 10.3390/diagnostics15222872

**Published:** 2025-11-13

**Authors:** Hasan Dogan, Ozkan Ozmuk, Zülfikar Memiş, Bilgehan Atilgan Acar, Emrah Aytac, Ferhat Balgetir, Aysenur Onalan, Ozlem Aykac, Zehra Uysal Kocabas, Muhammed Nur Ogun, Esra Ünal, Cetin Kursad Akpinar, Erdem Gurkas, Atilla Ozcan Ozdemir

**Affiliations:** 1 Department of Neurology, Faculty of Medicine, Samsun University, Samsun 55139, Turkey; 2 Department of Neurology, Prof. Dr. Cemil Tascioglu City Hospital, İstanbul 34384, Turkey; 3 Department of Neurology, Faculty of Medicine, Sakarya University, Sakarya 54100, Turkey; 4 Department of Neurology, Faculty of Medicine, Firat University, Elazığ 23119, Turkey; 5 Department of Neurology, Kartal Lutfi Kirdar Training and Research Hospital, University of Health Sciences, İstanbul 34732, Turkey; 6 Department of Neurology, Faculty of Medicine, Eskisehir Osmangazi University, Eskisehir 26040, Turkey; 7 Department of Neurology, Faculty of Medicine, Bolu Abant Izzet Baysal University, Bolu 14030, Turkey

**Keywords:** acute ischemic stroke, mechanical thrombectomy, C-reactive protein-to-lymphocyte ratio, prognosis, intracranial hemorrhage

## Abstract

**Background/Objectives:** The C-reactive protein-to-lymphocyte ratio (CLR) has emerged as an inflammatory biomarker reflecting innate and adaptive immune responses. Its prognostic value in acute ischemic stroke patients undergoing mechanical thrombectomy remains unclear. This study investigated whether CLR predicts functional outcome, mortality, and symptomatic intracranial hemorrhage (sICH). **Methods:** In this multicenter retrospective study, 714 patients with anterior circulation large-vessel occlusion treated with mechanical thrombectomy between January 2024 and January 2025 were analyzed. Clinical, angiographic, and laboratory data, including CLR, were collected. CLR was calculated as CRP divided by lymphocyte count/1000. Outcomes were 90-day modified Rankin Scale (mRS; poor outcome = 3–6; mortality = 6) and sICH per ECASS II. Receiver operating characteristic (ROC) analyses identified optimal CLR cut-offs. Logistic regression analyses determined independent predictors of outcomes. **Results:** sICH occurred in 39 patients (5.5%). CLR correlated with higher baseline NIHSS and lower ASPECTS. ROC analyses showed that CLR predicted poor functional outcome and mortality with an identical cut-off (≥7.51; AUCs 0.634 and 0.664), and demonstrated strong discrimination for sICH (cut-off ≥ 10.64; AUC 0.855). In multivariable analyses, CLR remained an independent predictor across all outcomes (ORs 1.02, 1.02, and 1.03, all *p* < 0.001), in addition to established clinical factors. **Conclusions:** Admission CLR was independently associated with poor outcomes, mortality, and sICH after mechanical thrombectomy. As an easily obtainable marker from routine laboratory tests, CLR may provide additional prognostic information and complement established predictors, but prospective validation is required.

## 1. Introduction

Acute ischemic stroke (AIS) triggers a systemic inflammatory response characterized by the release of proinflammatory cytokines and acute-phase reactants [1,2]. After the onset of ischemia, damage-associated molecular patterns (DAMPs) are released from the necrotic core [1]. These DAMPs trigger the production of proinflammatory cytokines such as IL-1β, IL-6, and TNF-α [2]. This inflammatory response weakens the blood–brain barrier and promotes the migration of neutrophils and lymphocytes into the injured tissue. The accumulation of reactive oxygen species (ROS) and proteases aggravates secondary neuronal injury and contributes to the expansion of the necrotic core [1,2].

Among biomarkers of systemic inflammation, C-reactive protein (CRP) has been widely used to assess stroke prognosis [3]. Elevated CRP levels consistently predict poor outcomes in AIS patients treated with intravenous thrombolysis or mechanical thrombectomy [4,5,6,7,8]. In parallel, increased sympathetic activation and hypothalamic–pituitary–adrenal axis stimulation after AIS reduce circulating lymphocyte counts, contributing to stroke-induced immunodepression [7,9,10]. Accordingly, lymphopenia has been associated with unfavorable clinical outcomes in acute stroke [10,11].

The C-reactive protein-to-lymphocyte ratio (CLR) has recently emerged as a novel biomarker that reflects both innate and adaptive immune responses [12]. Beyond reflecting systemic inflammation, CLR also mirrors stroke-induced immunodepression, both of which are critical determinants of post-stroke prognosis. Elevated CLR levels have been reported as predictors of poor outcomes and mortality in patients with COVID-19 [12], ulcerative colitis [13], and sepsis in intensive care units [14]. In cerebrovascular disease, one study reported that the lymphocyte-to-CRP ratio (LCR) predicted functional outcomes after aneurysmal subarachnoid hemorrhage (SAH) [15], whereas another demonstrated that high CLR values were associated with cerebral vasospasm following SAH [16].

Nevertheless, the available evidence on CLR in ischemic stroke is still very limited. To date, only one small single-center study has examined the lymphocyte-to-CRP ratio, reporting that low values were associated with futile recanalization after thrombectomy [11]. Whether CLR itself independently predicts functional outcomes, mortality, and hemorrhagic complications in patients undergoing mechanical thrombectomy remains uncertain.

Therefore, in this multicenter study including a large number of patients with anterior circulation stroke, we aimed to evaluate the prognostic value of admission CLR for predicting poor functional outcome, mortality, and symptomatic intracranial hemorrhage following mechanical thrombectomy.

## 2. Materials and Methods

### 2.1. Study Design and Participants

This retrospective multicenter cohort included consecutive patients with acute ischemic stroke due to anterior circulation large-vessel occlusion who underwent mechanical thrombectomy between January 2024 and January 2025 at seven tertiary stroke centers in Turkey. Eligible patients were ≥18 years of age, presented within 6 h of symptom onset, had a pre-stroke modified Rankin Scale (mRS) score of 0–1, demonstrated large-vessel occlusion on CT angiography, and had an Alberta Stroke Program Early CT Score (ASPECTS) > 5. Successful reperfusion was defined as achieving a modified Thrombolysis in Cerebral Infarction (mTICI) score of ≥2b.

Exclusion criteria were: (1) malignancy, (2) recent infection requiring treatment within the previous week, (3) severe metabolic disease, (4) advanced liver failure, (5) end-stage renal disease, (6) rheumatologic or hematologic disorders, (7) unsuccessful recanalization, and (8) incomplete data. Eligible patients presenting within 4.5 h of onset received intravenous recombinant tissue plasminogen activator (IV tPA) prior to thrombectomy. As this was a retrospective study, no formal sample size calculation was performed; all consecutive patients meeting the inclusion criteria during the study period were analyzed to maximize statistical power and minimize selection bias.

The study was approved by the local ethics committee and conducted in accordance with the Declaration of Helsinki. Owing to its retrospective design, the requirement for informed consent was waived.

### 2.2. Clinical, Angiographic, and Laboratory Data

Baseline demographic and clinical characteristics included age, sex, and vascular risk factors (hypertension, diabetes mellitus, hyperlipidemia, coronary artery disease, atrial fibrillation, smoking, and heart failure). Stroke severity at admission was assessed with the National Institutes of Health Stroke Scale (NIHSS), and baseline ischemic burden with ASPECTS.

Angiographic data included occlusion site, onset-to-groin puncture time, puncture-to-reperfusion time, number of thrombectomy passes, first-pass effect, distal embolization, and final mTICI score.

Venous blood samples were collected from all patients at the time of hospital admission, before the endovascular procedure. Biochemical analyses included complete blood count and serum C-reactive protein (CRP, mg/L). The C-reactive protein-to-lymphocyte ratio (CLR) was calculated as CRP divided by the lymphocyte count/1000.

### 2.3. Outcome Measures

The primary outcome was functional status at 90 days, assessed with the mRS. Favorable outcome was defined as mRS 0–2, poor outcome as mRS 3–6, and mortality as mRS 6. For descriptive purposes, baseline characteristics were presented in three groups (mRS 0–2, 3–5, and 6).

Intracerebral hemorrhage was assessed with a non-contrast CT performed within 24 h after thrombectomy. Symptomatic intracranial hemorrhage (sICH) was defined according to ECASS II criteria as any intracranial hemorrhage associated with neurological deterioration (≥4-point increase in NIHSS score).

### 2.4. Statistical Analysis

All analyses were performed using IBM SPSS Statistics version 23. The Kolmogorov–Smirnov test was used to assess normality. Continuous variables were expressed as mean ± standard deviation and median (minimum–maximum), and compared using the Mann–Whitney U test (two groups) or Kruskal–Wallis H test (≥3 groups). Categorical variables were reported as frequency (percentage) and compared using the Chi-square test or Fisher’s exact test, as appropriate.

Receiver operating characteristic (ROC) curve analysis with the Youden index was performed to determine optimal CLR cut-off values for predicting poor outcome, mortality, and sICH. The area under the curve (AUC) and 95% confidence intervals (CIs) were calculated.

Univariate logistic regression analyses were conducted for all candidate variables. Variables with a *p* value below 0.10 in the univariate analysis, along with those considered clinically important (such as age, sex, baseline NIHSS, and ASPECTS), were evaluated for possible inclusion in the multivariable logistic regression models. To maintain model stability and minimize collinearity, only predictors that contributed meaningfully to the overall model performance were retained in the final analysis. For the symptomatic intracranial hemorrhage (sICH) analysis, given the limited number of events (39 patients, 5.5%), variables with *p* < 0.05 in univariate analysis and clinically relevant covariates (age, sex, NIHSS, and ASPECTS) were included in the multivariable model.

To better understand whether CLR offers any extra predictive value beyond the well-known stroke predictors, we carried out additional statistical comparisons using the net reclassification improvement (NRI) and integrated discrimination improvement (IDI) indices. First, a base logistic model that included age, NIHSS, and ASPECTS was established. Then, CLR was added to form an extended model, and the two were compared. Continuous NRI and IDI estimates were generated with 500 bootstrap samples, from which 95% confidence intervals and *p*-values were derived.

Multicollinearity was evaluated using variance inflation factors (VIFs) and tolerance statistics; no significant collinearity was observed (all VIF < 5, tolerance > 0.2). Odds ratios (ORs) with 95% CIs were reported. A two-tailed *p*-value < 0.05 was considered statistically significant. Given the relatively low number of sICH events, Firth bias-reduced logistic regression was additionally performed to reduce small-sample bias. The detailed model outputs are provided in the Supplementary Materials.

## 3. Results

A total of 714 patients were included in the study, with a mean age of 66.2 ± 12.5 years (range: 19–96 years), and 54.1% (*n* = 386) were female. Symptomatic intracranial hemorrhage occurred in 39 patients (5.5%).

CLR showed a positive correlation with baseline NIHSS (ρ = 0.191; *p* < 0.001) and a negative correlation with ASPECTS (ρ = −0.150; *p* < 0.001). A weak positive correlation was also observed between symptom-to-recanalization time and CLR (ρ = 0.129; *p* < 0.001). No significant associations were identified between CLR and other clinical or demographic characteristics or vascular risk factors (all *p* > 0.05). In addition, CLR values did not differ significantly across stroke subtypes (*p* > 0.05).

When patients were stratified into three groups according to mRS (0–2, 3–5, and 6), CLR values are summarized in Table 1. CLR was markedly higher in the mRS 6 group, and the difference among the groups was statistically significant (*p* < 0.001).

### 3.1. Predictive Value of CLR (ROC Analyses)

**Figure 1:**
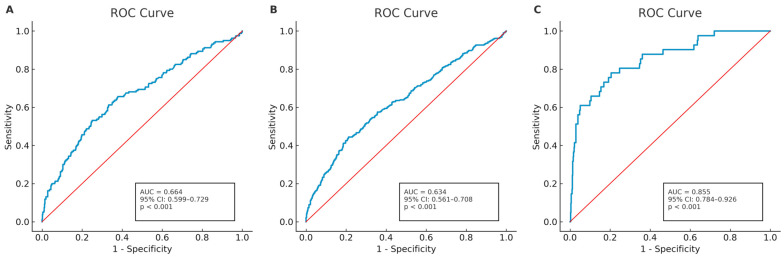
Receiver operating characteristic (ROC) curves showing the best cut-off value of CLR for different clinical outcomes: (**A**) mortality, (**B**) poor functional outcome, and (**C**) symptomatic intracranial hemorrhage (sICH).

Receiver operating characteristic (ROC) curve analysis identified a cut-off of ≥7.51 for predicting poor outcome (AUC 0.634; sensitivity 44.6%; specificity 78.6%) and mortality (AUC 0.664; sensitivity 52.5%; specificity 74.8%). For sICH, CLR demonstrated strong discrimination, with an optimal cut-off of ≥10.64 (AUC 0.855; sensitivity 78.1%; specificity 79.5%; *p* < 0.001) (Figure 1).

Because CLR was associated with functional outcomes in the main analyses, we further examined whether it could improve the performance of standard prognostic models. A base model containing age, NIHSS, and ASPECTS was compared with an extended version that also included CLR. The extended model showed a small but clear improvement in its ability to distinguish patients with poor outcomes (AUC 0.775 vs. 0.753; ΔAUC = 0.022; 95% CI 0.009–0.039; *p* < 0.001). Reclassification analyses supported these results, showing that adding CLR helped to more accurately classify patients by outcome risk. The continuous NRI was 0.317 (95% CI 0.171–0.456) and the IDI was 0.033 (95% CI 0.013–0.061), both statistically significant (*p* < 0.001) (Table 2).

### 3.2. Predictors of Poor Functional Outcome

In univariate analyses, age, NIHSS, ASPECTS, symptom-to-recanalization time, first-pass failure, distal embolism, diabetes mellitus, hypertension, hyperlipidemia, COPD, and CLR were significantly associated with poor outcome. In multivariate analysis, independent predictors included age, NIHSS, ASPECTS, first-pass failure, distal embolism, diabetes mellitus, and hyperlipidemia, with CLR remaining an independent predictor (OR: 1.02, *p* < 0.001) (Table 3).

### 3.3. Predictors of Mortality

In univariate analyses, age, NIHSS, ASPECTS, symptom-to-recanalization time, diabetes mellitus, hypertension, heart failure, coronary artery disease, COPD, and CLR were associated with mortality. In the multivariate analysis, independent predictors were NIHSS, ASPECTS, symptom-to-recanalization time, diabetes mellitus, heart failure and CLR (OR: 1.02; *p* < 0.001) (Table 4).

### 3.4. Predictors of sICH

In univariate analyses, age, NIHSS, ASPECTS, symptom-to-recanalization time, first-pass failure, and CLR were significantly associated with sICH. In the multivariate model, independent predictors included age, ASPECTS, first-pass failure, and CLR (OR: 1.03; *p* < 0.001) (Table 5).

Across all models, CLR consistently emerged as an independent predictor, while other predictors varied depending on the outcome.

## 4. Discussion

In this multicenter cohort of 714 patients undergoing mechanical thrombectomy for anterior circulation large-vessel occlusion, elevated admission CLR was independently associated with poor functional outcome, mortality, and sICH at 90 days (all *p* < 0.001). Although the odds ratios were modest, the associations were consistent across outcomes and statistically robust. CLR demonstrated strong discriminative ability for predicting sICH (AUC 0.855; cut-off ≥ 10.64), while the same threshold of ≥7.51 predicted both poor functional outcome and mortality. To the best of our knowledge, our study to date provides the first and only multicenter evidence showing that admission CLR is an independent predictor of poor functional outcome, mortality, and symptomatic intracranial hemorrhage in ischemic stroke patients undergoing mechanical thrombectomy.

In patients undergoing mechanical thrombectomy with successful recanalization, favorable clinical outcomes remain highly variable, ranging between 32% and 71% [17]. Numerous factors influence prognosis, and one that has received increasing attention is post-stroke inflammation. Following ischemic injury, the inflammatory response that begins in necrotic tissue is amplified by the release of proinflammatory cytokines, leading to systemic inflammation. Neutrophil infiltration, accumulation of reactive oxygen species, and the release of matrix metalloproteinases further contribute to blood–brain barrier disruption [5,7] Stroke also affects the autonomic nervous system: cytokines released from infarcted tissue can stimulate the hypothalamus and activate sympathetic pathways [18], resulting in increased catecholamine release [19], which in turn promotes immunodepression and lymphopenia [20]. Moreover, cytokine-driven activation of the hypothalamic–pituitary–adrenal axis elevates glucocorticoid secretion [21], and increased cortisol levels further contribute to lymphocyte depletion [22]. Together, these processes contribute to systemic inflammation and immune suppression, both of which may influence prognosis after stroke.

CRP is a hepatocyte-derived acute-phase protein induced primarily by interleukin-6 (IL-6). Its levels rise during inflammatory states [23]. After ischemic stroke, IL-6 elevation has been shown to correlate with increased CRP levels [24]. Hertog et al. reported that high CRP levels were associated with poor functional outcomes and mortality at three months in ischemic stroke patients [25]. Even when reperfusion was achieved with thrombolysis in middle cerebral artery occlusion, patients with high CRP levels demonstrated higher mortality [26]. In patients undergoing mechanical thrombectomy, studies have reported inconsistent results regarding the association between elevated CRP and poor outcomes or sICH: some demonstrated a significant relationship [4], whereas others did not [27]. In parallel, lymphopenia has consistently been associated with greater stroke severity and unfavorable clinical outcomes [28]. These findings support the concept that both elevated CRP and reduced lymphocyte counts reflect critical pathways influencing prognosis.

Several hematological indices have been investigated as potential prognostic biomarkers in ischemic stroke. Among these, the neutrophil-to-lymphocyte ratio (NLR) has been the most extensively studied. A recent meta-analysis confirmed its predictive value for poor functional outcome, symptomatic intracranial hemorrhage (sICH), and mortality in patients receiving reperfusion therapies [29]. Other ratios have also been evaluated; for example, in a thrombectomy cohort of 432 patients, the platelet-to-lymphocyte ratio (PLR) and platelet-to-neutrophil ratio (PNR) were associated with poor outcomes and mortality, while the platelet-to-white blood cell ratio (PWR) showed a significant ROC value for predicting functional outcome but was not an independent predictor in multivariable analysis [30]. The lymphocyte-to-monocyte ratio (LMR) was predictive of long-term functional outcome but not sICH [31]. More recently, elevated systemic immune-inflammation index (SII) values measured at 24 h have been reported to be associated not only with functional outcomes but also with sICH and mortality [32]. However, both the systemic inflammation response index (SIRI) and SII have been examined for their associations with functional outcomes, although their relationships with sICH and mortality were not investigated in another study [33]. Albumin-based ratios have also gained attention: the C-reactive protein-to-albumin ratio (CAR) has been linked to poor outcomes, mortality, and sICH [34], the fibrinogen-to-albumin ratio (FAR) was associated with futile recanalization [35], and the neutrophil percentage-to-albumin ratio (NPAR) was suggested to predict functional outcomes but showed no associations with sICH or mortality [36]. In another study, sex-related differences in inflammatory parameters were investigated between female and male populations [37]. Similarly, age-specific variations in inflammatory biomarkers and their impact on futile recanalization after mechanical thrombectomy have also been reported [38]. Overall, previous studies have focused on different outcome measures, with some evaluating only functional prognosis and others not addressing hemorrhagic complications or mortality. This heterogeneity highlights the need for more comprehensive biomarkers capable of capturing the full spectrum of outcomes in stroke patients.

CLR has recently been proposed as a novel biomarker integrating systemic inflammation and immune response. Elevated CLR has been associated with poor prognosis in conditions such as COVID-19 [39] and several malignancies, including pancreatic, colorectal, and lung cancers [40,41,42]. Evidence in cerebrovascular disease is limited. In a cohort of 166 patients with aneurysmal subarachnoid hemorrhage (SAH), Li et al. reported that high CLR levels were associated with cerebral vasospasm and delayed cerebral ischemia [16]. Similarly, Zhang et al. found that elevated CLR was linked to worse prognosis in aneurysmal SAH [15].

To date, among CRP–lymphocyte composite markers, only the lymphocyte-to-CRP ratio (LCR) has been evaluated in ischemic stroke patients undergoing mechanical thrombectomy, in a single-center cohort (*n* = 196). In that study, patients with futile recanalization had significantly lower LCR values than those with non-futile recanalization; however, LCR was not retained as an independent predictor in multivariable regression [11]. Although LCR and CLR are mathematically inverse transformations of the same CRP–lymphocyte construct, the prior study focused solely on futile recanalization and did not assess hemorrhagic complications or mortality. By contrast, our multicenter cohort extends these findings by showing that CLR provides independent prognostic information across a broader spectrum of clinically relevant outcomes, including poor functional outcome, mortality, and sICH.

In our analysis, separate ROC curves were generated for each clinical outcome, resulting in different optimal cut-off values for CLR. The small variations observed in CLR cut-off values across different outcomes may be related to distinct pathophysiological mechanisms involved after stroke. The discriminative ability of CLR was modest but consistent across outcomes, with an AUC of 0.634 for poor functional outcome and 0.664 for mortality. A prior study reported AUCs of 0.663 for PLR, 0.616 for PNR, and 0.583 for PWR [30]. Another study reported AUC values ranging from 0.606 to 0.682 for NLR, MLR, PLR, SII, and SIRI [32], while CAR has reached approximately 0.72 in some cohorts [33]. Taken together, these findings indicate that the predictive performance of CLR in our study aligns well with existing literature and reflects the multifactorial nature of stroke recovery.

When compared with other blood-based ratios studied in stroke, such as the NLR or the CAR, the CLR seems to offer a broader view of inflammation. NLR primarily reflects the leukocyte-mediated inflammatory response, while CAR reflects the acute-phase reaction. In contrast, CLR combines both the inflammatory activity reflected by CRP and the immune suppression indicated by lymphocyte counts. However, its predictive power for overall outcome and mortality was modest. Therefore, we also examined whether CLR adds extra value to standard predictors such as age, NIHSS, and ASPECTS. Adding CLR slightly but significantly improved model performance, producing higher AUC values and significant NRI and IDI results. These findings suggest that CLR may provide additional benefit when used alongside other established predictors in assessing functional outcomes after thrombectomy. Although the improvement was small, this is typical for single biomarkers in a complex disease such as stroke. Because CLR is inexpensive and readily available, it may serve as a practical supporting marker for early outcome prediction.

This study has several limitations. Its retrospective design makes it difficult to establish causal relationships and carries a potential risk of missing or incomplete data. Moreover, patients with active malignancy, recent infection, or severe systemic diseases were excluded, which may reduce the generalizability of our findings to the broader stroke population. The analysis focused exclusively on anterior circulation large-vessel occlusions, and, therefore, the results may not be directly applicable to posterior circulation strokes. Although our study evaluated outcomes in patients who underwent thrombectomy, generalization to small-vessel stroke populations is limited because such patients were not included. Another limitation is the relatively small number of symptomatic intracranial hemorrhage events (*n* = 39), which limits the statistical power of this specific analysis and could increase the risk of model overfitting, although we carefully assessed collinearity and model calibration to minimize this concern. Finally, CRP and lymphocyte values were measured only once at admission, without serial follow-up, making it impossible to evaluate temporal changes in the inflammatory response.

## 5. Conclusions

In the early phase, elevated CLR or decreased LCR may provide prognostic information regarding poor clinical outcomes, mortality, and symptomatic intracranial hemorrhage in patients undergoing mechanical thrombectomy. Its prognostic value should be further validated in large-scale, prospective, randomized controlled trials.

## Figures and Tables

**Table 1:** Baseline characteristics and CLR values stratified by mRS groups.

Variable	mRS 0–2 (*n* = 411)	mRS 3–5 (*n* = 145)	mRS 6 (*n* = 158)	*p*-Value
Age (mean ± SD)	64.08 ± 13.18	68.74 ± 10.66	69.26 ± 10.91	**<0.001**
Female (%)	54.0%	52.4%	55.7%	0.848
NIHSS (median, min–max)	14.0 (2–24)	16.0 (5–25)	18.0 (5–32)	**<0.001**
ASPECTS (median, min–max)	9.0 (6–10)	8.0 (5–10)	8.0 (6–10)	**<0.001**
IV-tPA (%)	31.6%	30.3%	31.0%	0.957
Symptom-to-recanalization (min, median [IQR])	285 [240–340]	310 [260–365]	325 [270–380]	**<0.001**
First-pass (%)	43.1%	31.0%	31.6%	**<0.001**
Number of passes (median, min–max)	2.0 (1–15)	2.0 (1–7)	2.0 (1–8)	**<0.001**
Distal emboli (%)	17.5%	27.6%	20.3%	**0.034**
Symptomatic hemorrhage (%)	2.0%	5.5%	9.0%	**<0.001**
CLR (mean ± SD)	7.00 ± 13.30	12.52 ± 26.49	25.56 ± 49.53	**<0.001**

Values are presented as mean ± SD, median (min–max) or percentage (%). CLR: C-reactive protein to lymphocyte ratio; IV-tPA: intravenous tissue plasminogen activator; mRS: modified Rankin Scale. Bold values indicate statistical significance (*p* < 0.05).

**Table 2:** Incremental prognostic value of CLR for poor functional outcome based on NRI and IDI analyses.

Metric	Estimate	95 CI	*p*-Value (Bootstrap)
AUC (Base model)	0.753	–	–
AUC (Extended model)	0.775	–	–
ΔAUC (Extended − Base)	0.022	0.009–0.039	**<0.001**
Continuous NRI	0.317	0.171–0.456	**<0.001**
IDI	0.033	0.013–0.061	**<0.001**

Base model: age + NIHSS + ASPECTS; Extended model: base + CLR, AUC: area under the curve; CI: confidence interval; NRI: net reclassification improvement; IDI: integrated discrimination improvement. Bold values indicate statistical significance (*p* < 0.05).

**Table 3:** Univariate and multivariate predictors of poor functional outcome (mRS 3–6).

Variable	Univariate OR (95% CI)	*p* Value (Univariate)	Multivariate OR (95% CI)	*p* Value (Multivariate)
Age, years	1.03 (1.02–1.05)	**<0.001**	1.03 (1.02–1.05)	**<0.001**
Male (ref: Female)	1.00 (0.74–1.34)	0.977	1.17 (0.81–1.69)	0.382
NIHSS at baseline	1.17 (1.13–1.22)	**<0.001**	1.15 (1.11–1.20)	**<0.001**
ASPECTS	0.60 (0.53–0.68)	**<0.001**	0.67 (0.57–0.78)	**<0.001**
Symptom-to-recanalization, min	1.00 (1.00–1.01)	**<0.001**	1.00 (1.00–1.00)	0.106
First-pass success	0.60 (0.44–0.82)	**0.002**	0.56 (0.38–0.81)	**0.002**
Distal emboli	1.47 (1.02–2.12)	**0.040**	1.96 (1.26–3.05)	**0.003**
IV-tPA	0.96 (0.69–1.32)	0.789	0.94 (0.63–1.40)	0.785
Diabetes mellitus	1.89 (1.37–2.60)	**<0.001**	1.83 (1.23–2.71)	**0.003**
Hypertension	1.40 (1.02–1.92)	**0.035**	0.93 (0.63–1.39)	0.728
Atrial fibrillation	1.03 (0.76–1.39)	0.861	-	-
Hyperlipidemia	0.65 (0.48–0.88)	**0.005**	0.56 (0.38–0.80)	**0.002**
Heart failure	1.40 (0.96–2.05)	0.084	-	-
Coronary artery disease	1.20 (0.88–1.63)	0.246	-	-
COPD	2.03 (1.24–3.30)	**0.005**	1.42 (0.77–2.59)	0.253
CLR	1.03 (1.02–1.04)	**<0.001**	1.02 (1.01–1.03)	**<0.001**

Values are presented as mean ± SD, median (min–max) or percentage (%). mRS: modified Rankin Scale; NIHSS: National Institutes of Health Stroke Scale; ASPECTS: Alberta Stroke Program Early CT Score; IV-tPA: intravenous tissue plasminogen activator; COPD: chronic obstructive pulmonary disease; CLR: C-reactive protein-to-lymphocyte ratio. Bold values indicate statistical significance (*p* < 0.05).

**Table 4:** Univariate and multivariate predictors of mortality (mRS 6).

Variable	Univariate OR (95% CI)	*p* Value (Univariate)	Multivariate OR (95% CI)	*p* Value (Multivariate)
Age, years	1.03 (1.01–1.05)	**<0.001**	1.01 (1.00–1.03)	0.114
Male (ref: Female)	0.92 (0.64–1.31)	0.640	1.01 (0.66–1.54)	0.959
NIHSS at baseline	1.21 (1.16–1.26)	**<0.001**	1.19 (1.13–1.25)	**<0.001**
ASPECTS	0.62 (0.54–0.71)	**<0.001**	0.76 (0.64–0.90)	**0.002**
Symptom-to-recanalization, min	1.01 (1.00–1.01)	**<0.001**	1.01 (1.00–1.01)	**0.002**
First-pass success	0.70 (0.48–1.01)	0.059	0.66 (0.42–1.03)	0.067
Distal emboli	1.01 (0.65–1.56)	0.976	1.13 (0.68–1.90)	0.623
IV-tPA	0.99 (0.67–1.45)	0.946	0.86 (0.54–1.38)	0.550
Diabetes mellitus	1.97 (1.37–2.84)	**<0.001**	1.72 (1.1–2.69)	**0.017**
Hypertension	1.50 (1.02–2.21)	**0.039**	1.06 (0.66–1.72)	0.785
Atrial fibrillation	1.08 (0.76–1.54)	0.677	-	-
Hyperlipidemia	0.76 (0.53–1.10)	0.144	0.66 (0.43–1.02)	0.063
Heart failure	2.31 (1.52–3.49)	**<0.001**	2.26 (1.36–3.77)	**0.002**
Coronary artery disease	1.45 (1.01–2.07)	**0.044**	-	-
COPD	2.39 (1.44–3.97)	**<0.001**	-	-
CLR	1.02 (1.01–1.03)	**<0.001**	1.02 (1.01–1.03)	**<0.001**

Abbreviations: OR: odds ratio; CI: confidence interval; mRS: modified Rankin Scale; NIHSS: National Institutes of Health Stroke Scale; ASPECTS: Alberta Stroke Program Early CT Score; IV-tPA: intravenous tissue plasminogen activator; COPD: chronic obstructive pulmonary disease; CLR: C-reactive protein-to-lymphocyte ratio. Mortality defined as mRS = 6 at 90 days. Univariate models were fitted separately for each covariate. The multivariate model included all listed covariates. Bold values indicate statistical significance (*p* < 0.05).

**Table 5:** Univariate and multivariate predictors of symptomatic intracranial hemorrhage (sICH).

Variable	Univariate OR (95% CI)	*p* Value (Univariate)	Multivariate OR (95% CI)	*p* Value (Multivariate)
Age, years	1.05 (1.01–1.08)	**0.008**	1.05 (1.01–1.09)	**0.010**
Male (ref: Female)	1.25 (0.66–2.39)	0.492	1.42 (0.66–3.04)	0.364
NIHSS at baseline	1.11 (1.03–1.18)	**0.003**	1.06 (0.99–1.14)	0.081
ASPECTS	0.60 (0.47–0.76)	**<0.001**	0.68 (0.51–0.91)	**0.009**
Symptom-to-recanalization, min	1.01 (1.00–1.01)	**0.004**	-	-
First-pass success	0.40 (0.18–0.89)	**0.024**	0.24 (0.09–0.67)	**0.007**
Distal emboli	1.02 (0.46–2.28)	0.956	-	-
IV-tPA	0.86 (0.42–1.76)	0.675	-	-
Diabetes mellitus	1.22 (0.62–2.40)	0.558	-	-
Hypertension	1.63 (0.78–3.40)	0.192	-	-
Atrial fibrillation	1.03 (0.54–1.98)	0.924	-	-
Hyperlipidemia	0.71 (0.36–1.39)	0.315	-	-
Heart failure	1.84 (0.89–3.79)	0.101	-	-
Coronary artery disease	0.85 (0.43–1.68)	0.641	-	-
COPD	1.99 (0.84–4.67)	0.116	-	-
CLR	1.03 (1.02–1.04)	**<0.001**	1.03 (1.02–1.04)	**<0.001**

Abbreviations: OR: odds ratio; CI: confidence interval; mRS: modified Rankin Scale; NIHSS: National Institutes of Health Stroke Scale; ASPECTS: Alberta Stroke Program Early CT Score; IV-tPA: intravenous tissue plasminogen activator; COPD: chronic obstructive pulmonary disease; CLR: C-reactive protein-to-lymphocyte ratio. Univariate models were fitted separately for each covariate. The multivariate model included all listed covariates. Bold values indicate statistical significance (*p* < 0.05).

## Data Availability

Due to the nature of the research, for ethical reasons supporting data is not available.

## Supplementary Materials

The following supporting information can be downloaded at: https://www.mdpi.com/article/10.3390/diagnostics15222872/s1, Table S1: Results of Firth Bias-Reduced Logistic Regression Analysis for Symptomatic Intracranial Hemorrhage.
