# Supplementary material for: C-Reactive Protein-to-Lymphocyte Ratio as a Prognostic Biomarker in Acute Ischemic Stroke Patients Undergoing Mechanical Thrombectomy: A Multicenter Study
Source: Diagnostics (Basel). 2025 Nov 13;15(22):2872. doi: 10.3390/diagnostics15222872 (PMC12651082; doi:10.3390/diagnostics15222872)
Supplement: Supplementary file 1 [file diagnostics-15-02872-s001.zip › diagnostics-3918972-supplementary.pdf]

Supplementary Table S1.

Results of Firth Bias-Reduced Logistic Regression Analysis for Symptomatic Intracranial Hemorrhage

**Variable**	**OR**	**95% CI**	**p**
**Age**	1.05	1.02 – 1.09	0.010
**Sex (1 = Male)**	1.41	0.92 – 2.28	0.110
**NIHSS**	1.07	1.02 – 1.13	0.021
**ASPECTS**	0.69	0.53 – 0.90	0.009
**First-pass**	0.26	0.09 – 0.67	0.007
**CLR**	1.026	1.01 – 1.04	<0.001

Note: OR = odds ratio; CI = confidence interval; CLR= C-reactive protein-to-lymphocyte ratio; NIHSS= National Institutes of Health Stroke Scale; ASPECTS= Alberta Stroke Program Early CT Score
